# Bronchial Remodeling Following Airway Stenting in Pediatric Patients With Tracheobronchial and Congenital Heart Disease

**DOI:** 10.1016/j.jscai.2023.101068

**Published:** 2023-07-11

**Authors:** Howaida El-Said, Katherine Price, Amira Hussein, Srujan Ganta, Aparna Rao, John Nigro, Matthew T. Brigger

**Affiliations:** a Pediatric Cardiology, Rady Children’s Hospital, San Diego, California; b University of California San Diego School of Medicine, San Diego, California; c Cardiothoracic Surgery, Rady Children’s Hospital, San Diego, California; d Pediatric Pulmonology, Rady Children’s Hospital, San Diego, California; e Pediatric Otolaryngology, Rady Children’s Hospital, San Diego, California; f Department of Otolaryngology-Head and Neck Surgery, University of California San Diego, San Diego, California

**Keywords:** airway stent, bronchial remodeling, congenital heart disease, tracheobronchial disease

## Abstract

**Background:**

Treatment of tracheobronchial disease in medically complex infants with congenital heart disease (CHD) is often challenging. When conservative management or surgery fails or is contraindicated, airway stenting can allow for advancement of care or weaning of respiratory support.

**Methods:**

We identified 8 cases of airway stenting with balloon-expandable coronary bare-metal stents performed at our institution between February 2019 and September 2022 to relieve conservative treatment-refractory tracheobronchial disease in pediatric patients with CHD. All patients underwent rigid microlaryngoscopy, bronchoscopy, and flexible bronchoscopy as well as computed tomography angiography.

**Results:**

Eight patients underwent technically uncomplicated placement of balloon-expandable coronary bare-metal stents in the trachea or bronchus. Immediate improvement in respiratory parameters was noted following stent placement. Six patients were able to wean mechanical ventilation following stent placement, with a median of 2.5 days of mechanical ventilation following the procedure (range, 0-219). All stents were subsequently endoscopically removed at a median of 6.8 months (range, 0.4-16.3 months). In 6 patients, bronchoscopy after stent removal demonstrated a rounder configuration of the airway consistent with bronchial remodeling.

**Conclusions:**

In pediatric patients with tracheobronchial and CHD, airway stenting with balloon-expandable bare-metal coronary stents relieved respiratory symptoms with minimal complications and resulted in bronchial remodeling after stent removal.

## Highlights

• Balloon-expandable coronary bare-metal stents can be used for bronchial stenting.
• Bronchial stenting can allow for advancement of care in complex cardiac patients.
• Stenting may result in bronchial remodeling apparent after stent removal.

## Central Illustration

**Figure:**
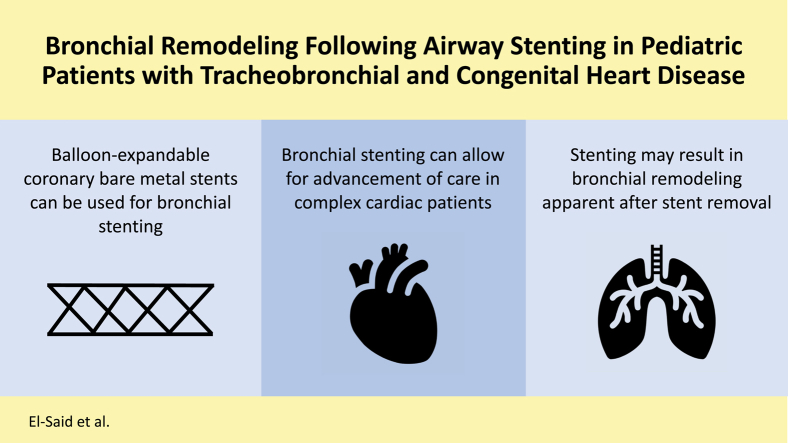

## Introduction

Treatment of tracheobronchial disease in infants can be challenging, particularly when it is caused by or concurrent with other comorbidities, most commonly vascular anomalies.^1^^,^^2^ When conservative management or definitive surgery fails or is contraindicated, airway stenting can provide a unique therapeutic option that allows for advancement of care. Intraluminal airway stenting has been used extensively in adults, but the pediatric airway presents distinctive challenges that have slowed clinical application. Pediatric tracheobronchial tissue is inherently weaker, making erosion more likely,^3^ and the benign nature of most pediatric tracheobronchial disease means the stent itself must have adequate radial force to remain in place but also have a low risk of harm. Additionally, unlike adults, expected pediatric airway growth requires the ability for progressive increases in stent size^3^ or ease of removal. For this reason, balloon-expandable bare-metal stents represent an adaptable treatment that can be expanded with the growth of the child and allow them to wean respiratory support or progress in their care.^4^^,^^5^ Here we report our outcomes in pediatric patients with tracheobronchial and congenital heart disease (CHD) who underwent balloon-expandable bare-metal stent placement for treatment-refractory tracheobronchial disease, which resulted in remodeling of the bronchus upon stent removal.

## Methods

### Patients

We identified 8 consecutive cases of airway stenting performed at our institution between February 2019 and September 2022 to relieve conservative treatment-refractory tracheobronchial disease in patients with concurrent CHD. All patients underwent rigid microlaryngoscopy, bronchoscopy, and flexible bronchoscopy to evaluate the airway as well as computed tomography angiography. This study was approved by the University of California Institutional Review Board (#180336).

### Procedure/technique

**Figure 1:**
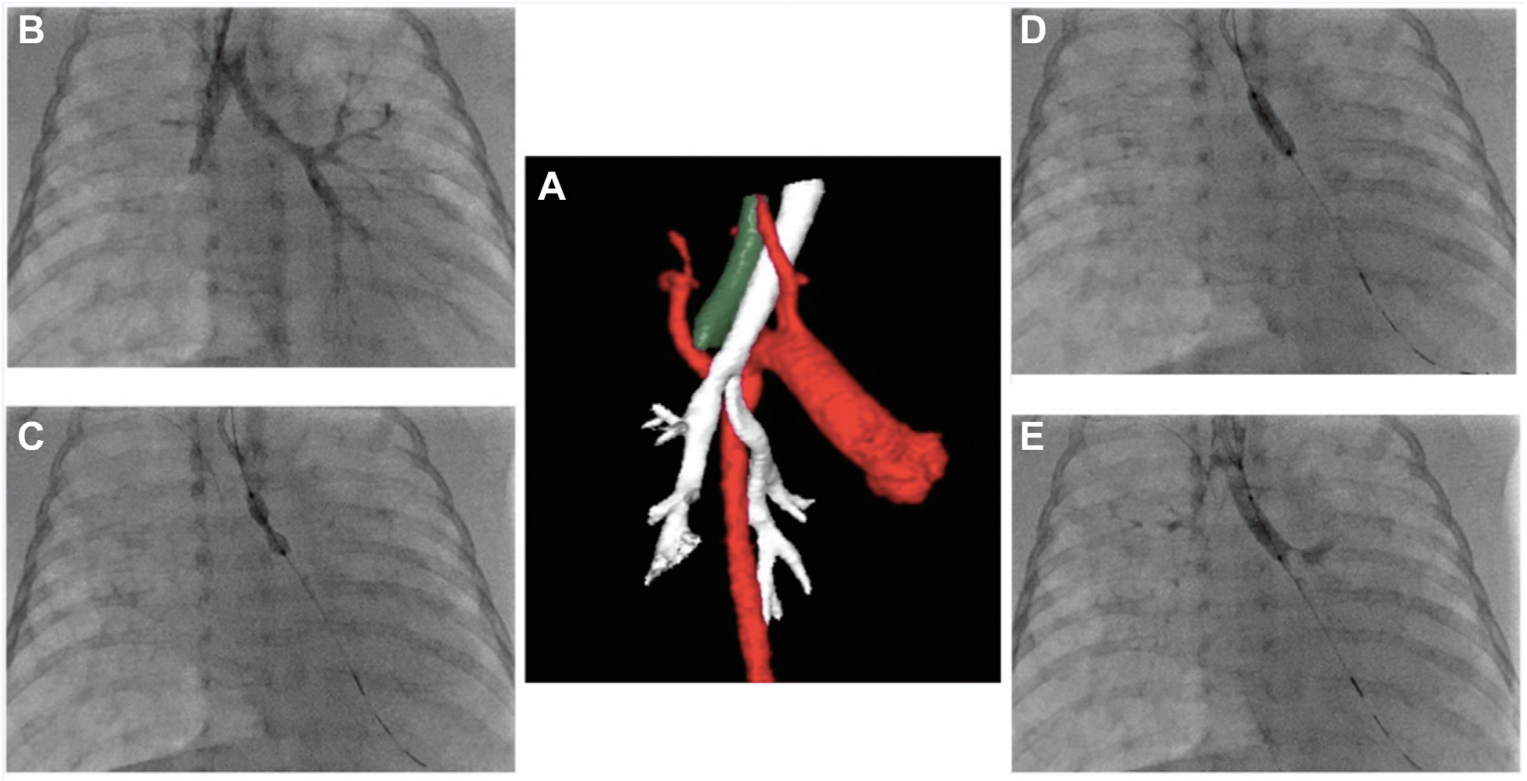
**Procedural steps.** (**A**) Three-dimensional segmentation of computed tomography angiogram showing the compressed bronchus in white, aorta in red, and esophagus in dark green. (**B**) Bronchogram showing the area of narrowing of the distal left bronchus at the take-off of the upper and lower branches. (**C, D**) Showing balloon inflation of the stent; note the narrowing midstent. (**E**) Bronchogram after stent placement showing improved caliber of the bronchus.

**Figure 2:**
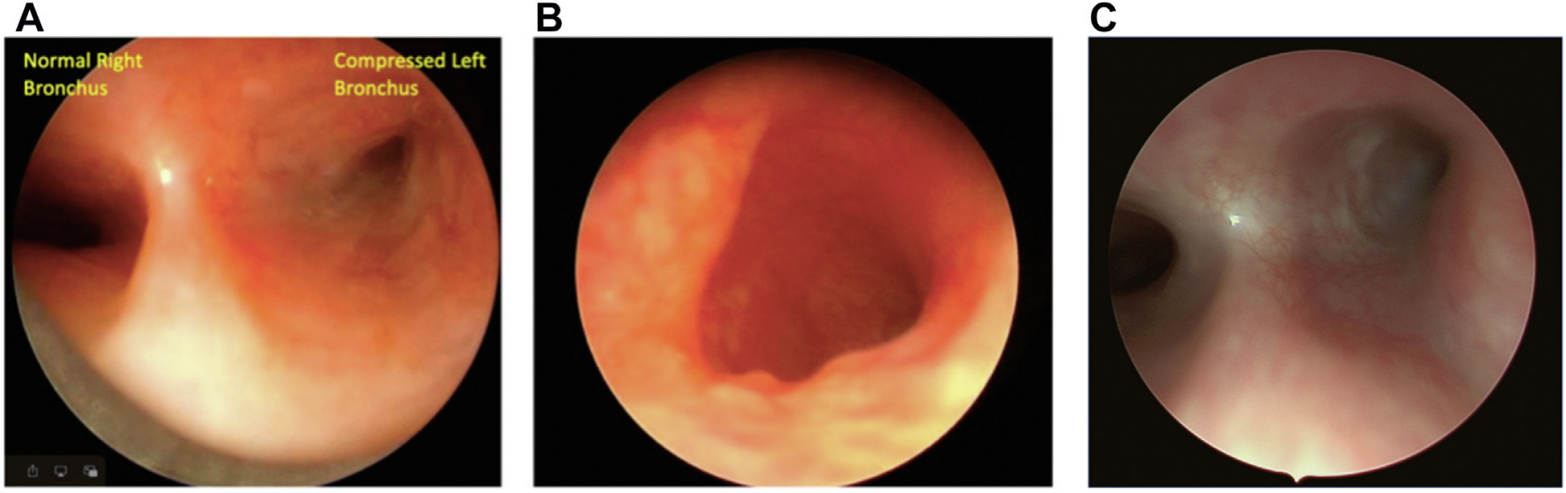
**Marked improvement of the right bronchus after stenting because of remodeling.** (**A**) Before stenting: left bronchus narrowed secondary to compression. (**B**) Six weeks after stent removal: left bronchus round. (**C**) One year after stent removal: sustained remodeling.

Biplane x-ray fluoroscopy was utilized in the cardiac catheterization laboratory (Infinix *I*; Toshiba) to perform airway stenting under general anesthesia. Before stenting, flexible bronchoscopy and 3-dimensional rotational angiography (Vitrea; ViTAL) were performed for a baseline evaluation of the bronchus and adjacent structures (Figure 1A, B). A bronchoscope swivel adapter was placed on the endotracheal tube, and an angled glide catheter (Terumo) with a Wholey wire (Medtronic) was inserted with the tip just distal to the endotracheal tube. Using this catheter system, biplane contrast bronchography was performed using 2 mL of diluted iodinated contrast (1 mL of contrast [Optiray 320 Ioversol Injection 68%] and 4 mL of normal saline), and positive-pressure breaths were administered to opacify the bronchial tree. The contrast was then suctioned out through the same catheter. Initial experience indicated that predilation was not required for accurate stent placement because these were low resistance lesions regardless of whether disease was caused by external compression or primary airway pathology. Minimal luminal diameter, proximal reference diameter, and distal reference diameter of the bronchus were measured, and stent size was chosen with a stent diameter equivalent to or 1 mm larger than the distal bronchus diameter. Stent length was chosen to cover the narrowing of the airway and avoid the proximal carina and distal bronchus bifurcation. The angled glide catheter and Wholey wire were advanced past the lesion and into a lower bronchial branch, and the Wholey wire was then exchanged for a 0.014” guidewire (Thruway, Boston Scientific). Using this system, an Integrity bare metal stent (Medtronic) or a Visi-Pro EV3 stent was deployed under fluoroscopic guidance (Figure 1C, D). Flexible and rigid bronchoscopy was used intermittently throughout the procedure to provide direct visualization (Figure 2). Repeat dilute-contrast bronchographies were performed to confirm stent position and stent apposition to the bronchus (Figure 1E). Periodic bronchoscopy was performed for surveillance. Stent removal was performed under direct bronchoscopic visualization using optical forceps. Removal was followed by self-limited minor mucosal bleeding in all cases.

## Results

Eight infants underwent stenting and subsequent stent removal. Seven received an Integrity bare metal coronary stent (Medtronic) and 1 received a Visi-Pro EV3 bare-metal stent. Patient demographics and clinical courses are described in Table 1. The median age and weight at stent placement were 4.04 months (range, 1.6-19.3 months) and 5.9 kg (range, 3.4-11.7 kg), respectively. Three of 8 patients had identified genetic syndromes, 6/8 have had cardiac surgery, and 2 of 8 had undergone tracheal surgery (tracheal resection and laryngotracheal reconstruction in one child and a slide tracheoplasty in another). Stents were not placed across sites of airway surgery. Four of 8 patients required tracheostomy at some point in their medical care: 1 because of tracheomalacia (remote from site of bronchial stent) while the stent was in place and 3 before stent placement. None required tracheostomy placement after stent removal.

**Table 1:** Patient demographics and clinical course.

Patient	Cardiac diagnosis	Genetic diagnosis	Airway anomalies	Reason for stent placement	Age at stent	Stent size and type	Stent location	Clinical result after stent placement	Time after stent implantation to stent removal	Reason for stent removal.	Bronchoscopy findings during follow-up
1	Heterotaxy, CAVC, ventricular inversion, pulmonary atresia	None	Tracheobronchomalacia	Unable to wean positive-pressure ventilation because of bronchial compression while awaiting optimal timing of cardiac repair	2.4 mo	5 × 9 mm Integrity bare metal stent	Right mainstem bronchus	Weaned to simple nasal cannula and discharged for home. Several months later, the patient underwent cardiac repair but, soon after, required tracheostomy because of tracheomalacia where the aorta crossed (not at site of bronchial stent)	11.8 mo	Symptoms improved. Status post cardiac repair and tracheostomy decannulation.	13 mo after stent removal: distal tracheomalacia where aorta crosses, right bronchus with minimal stenosis and no dynamic collapse at the site of previous stent
2	Truncus arteriosus type A3	None	Tracheobronchomalacia secondary to enlarged pulmonary artery	Despite previous cardiac repair, bronchial compression from enlarged pulmonary artery continued, requiring increasingly higher levels of respiratory support. Stent attempted to avoid immediate need for surgical pulmonary arteriopexy	18.8 mo	4 × 15 mm Integrity bare metal stent	Left bronchus with the proximal end at the carina	Weaned to room air within a few days of stent placement	8.9 mo	Symptoms improved. Stent removed at the time of later pulmonary artery reconstruction. Mild granulation tissue was found in stent at the time of removal.	5 mo after stent removal: scarring in the region of stenting requiring lysis of adhesions. 9 mo after stent removal: completely effaced bronchus at the previous site of stent requiring PA plasty.
3	Tetralogy of Fallot (absent pulmonary valve)	DiGeorge syndrome	Narrowing of the left mainstem bronchus	Unable to wean positive-pressure ventilation after cardiac surgery (bronchial compression slightly improved but still significant)	9.6 mo	4 × 15 mm Integrity bare metal stent	Left mainstem bronchus	Weaned to simple nasal cannula and discharged for home	16.3 mo	Symptoms improved.	4 mo after stent removal: good bronchial remodeling at site of previous stent placement, mild narrowing in second mainstem bronchus.
4	TAPVR	None	Multilevel airway stenosis and malacia	Multiple failed extubations because of dynamic collapse at previous tracheostomy site after tracheostomy decannulation and tracheal resection and laryngotracheal reconstruction	19.3 mo	9 × 17 mm Visi-Pro EV3 bare metal stent	Trachea	Temporary tracheal stenting allowed weaning to high-flow nasal cannula	11 d (0.4 mo)	At short-term follow-up bronchoscopy, the stent had distally migrated to the previous site of anastomosis. The site of collapse where the stent was originally placed showed only mild stenosis, so the stent was removed, and patient was weaned to room air.	4 mo after stent removal: well-healed tracheal resection site, mild tracheal stenosis.
5	DORV	DiGeorge syndrome	Left mainstem bronchus narrowing	After tracheostomy, continued to require sedation and paralysis with positive-pressure and heliox because of severe bronchomalacia	3.3 mo	4 × 12 mm Integrity bare metal stent	Left mainstem bronchus	Weaned paralytic and heliox within a few days, then optimized nutrition and vent settings before cardiac surgery. Weaned to only nighttime ventilator dependence by the time of discharge	3.7 mo	Symptoms improved, tolerated tracheostomy capping, status post cardiac repair.	1 mo after stent removal: mild bronchomalacia, subglottic dilation performed. 2 mo after stent removal: mild bronchomalacia, subglottic dilation performed. 3 mo after stent removal: mild bronchomalacia, closure of tracheocutaneous fistula.
6	DORV	None	Compression of the left mainstem bronchus	Unable to wean positive-pressure ventilation after cardiac surgery	4.8 mo	3.5 × 12 mm Integrity bare metal stent	Left mainstem bronchus	Weaned to room air within a few days of stent placement	5.1 mo	Symptoms improved. Occlusive granuloma noted at the time of stent removal.	1 mo after stent removal: mild bronchomalacia with remodeling at site of stent placement
7	Vascular ring, aberrant right subclavian artery	None	Bronchial narrowing at the level of a vascular ring in the setting of right lung agenesis and complete tracheal rings	Multiple failed extubations after slide tracheoplasty because of dynamic collapse at the level of a vascular ring distal to the tracheoplasty repair	2.9 mo	5 × 9 mm Integrity bare metal stent	Left mainstem bronchus	Successfully extubated within a few days of stent placement. Weaned to simple nasal cannula by time of discharge	8.5 mo	Symptoms improved; however, after planned stent removal 8 mo after placement, patient was again unable to be extubated because of continued vascular ring (aortic) compression. A new stent was placed at the same site and patient weaned to simple nasal cannula by the time of discharge. 3 wk later, the patient underwent aortopexy and placement of external splint on bronchus, with removal of intraluminal bronchial stent, and weaned to simple nasal cannula by the time of discharge.	5 d after (second) stent removal: left bronchus healing well, patent, with minimal malacia. 1.5 mo after (second) stent removal: left bronchus with mild bronchomalacia
8	DORV	Trisomy 18	Severe tracheobronchomalacia	High ventilatory requirements and work of breathing on tracheostomy. Palliative stent considered as family’s goal was to discharge for home	1.6 mo	Two 4 ×15 mm Integrity bare metal stents	Left mainstem bronchus	Decreased level of ventilatory support, transitioned to Astral ventilator at time of discharge home	4.2 mo	Symptoms improved; however, after removal of both stents, patient had partial left lung collapse with poor gas exchange and increased work of breathing.	10 d after stent removal: severe left bronchomalacia. Two new overlapping stents placed (5 mm × 20 mm and 5 mm × 16 mm Megatron), which improved ventilation and remain in place currently (3 mo since placement).

Pediatric patients who underwent airway stenting at Rady Children’s Hospital in San Diego between February 2019 and September 2022.

CAVC, complete atrioventricular canal; DORV, double outlet right ventricle; PA, pulmonary artery; TAPVR, total anomalous pulmonary venous return.

Most patients had severe bronchomalacia with acute life-threatening events requiring sedation and paralysis, which improved immediately after bronchial stent placement. Seven patients underwent placement of a single stent, and 1 patient underwent placement of 2 stents during the same procedure to adequately cover the compressed portion of the bronchus. The median fluoroscopy time was 6.34 minutes (range, 0.7-16.5 minutes). Six of 8 patients were able to wean mechanical ventilation following stent placement, with a median of 2.5 days of mechanical ventilation following the procedure (range, 0-219 days). Three of 8 patients required additional airway procedures after stent placement, although only one involved the site of stenting. No balloon dilations of stents were performed. Two of 8 patients underwent cardiac surgery following stent placement to relieve airway compromise.

**Figure 3:**
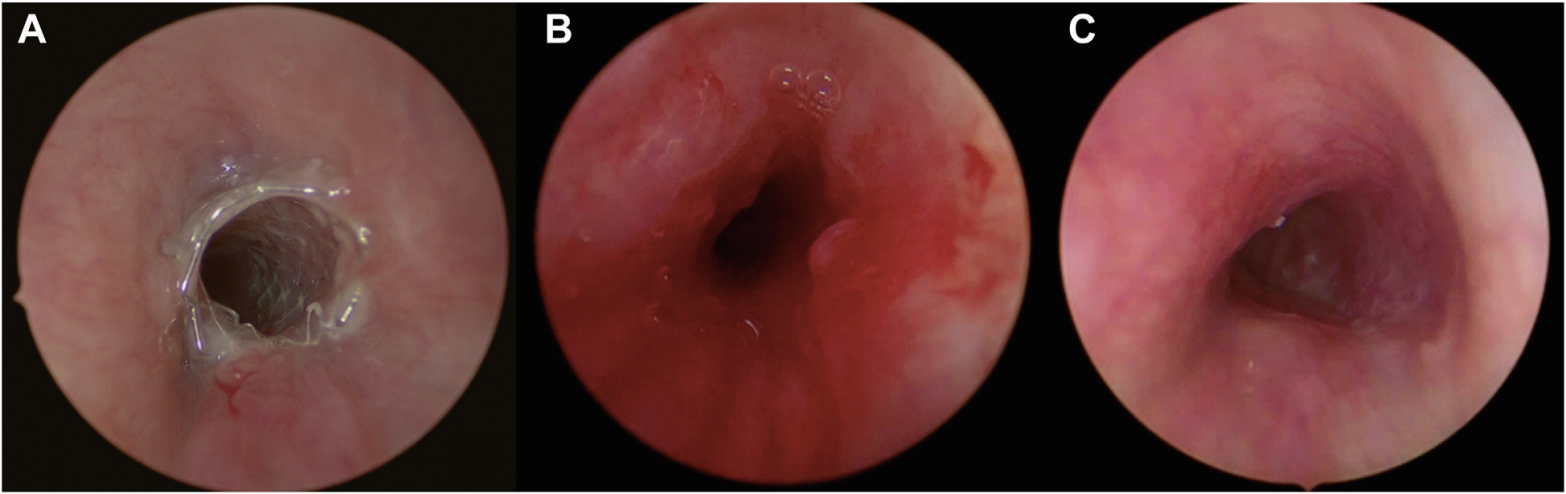
**Stent removal and remodeling.** (**A**) Stent in left bronchus just prior to removal. (**B**) Bronchus immediately after removal with minor self-limited bleeding. (**C**) Bronchus with retained remodeled shape 6 months after removal.

**Figure 4:**
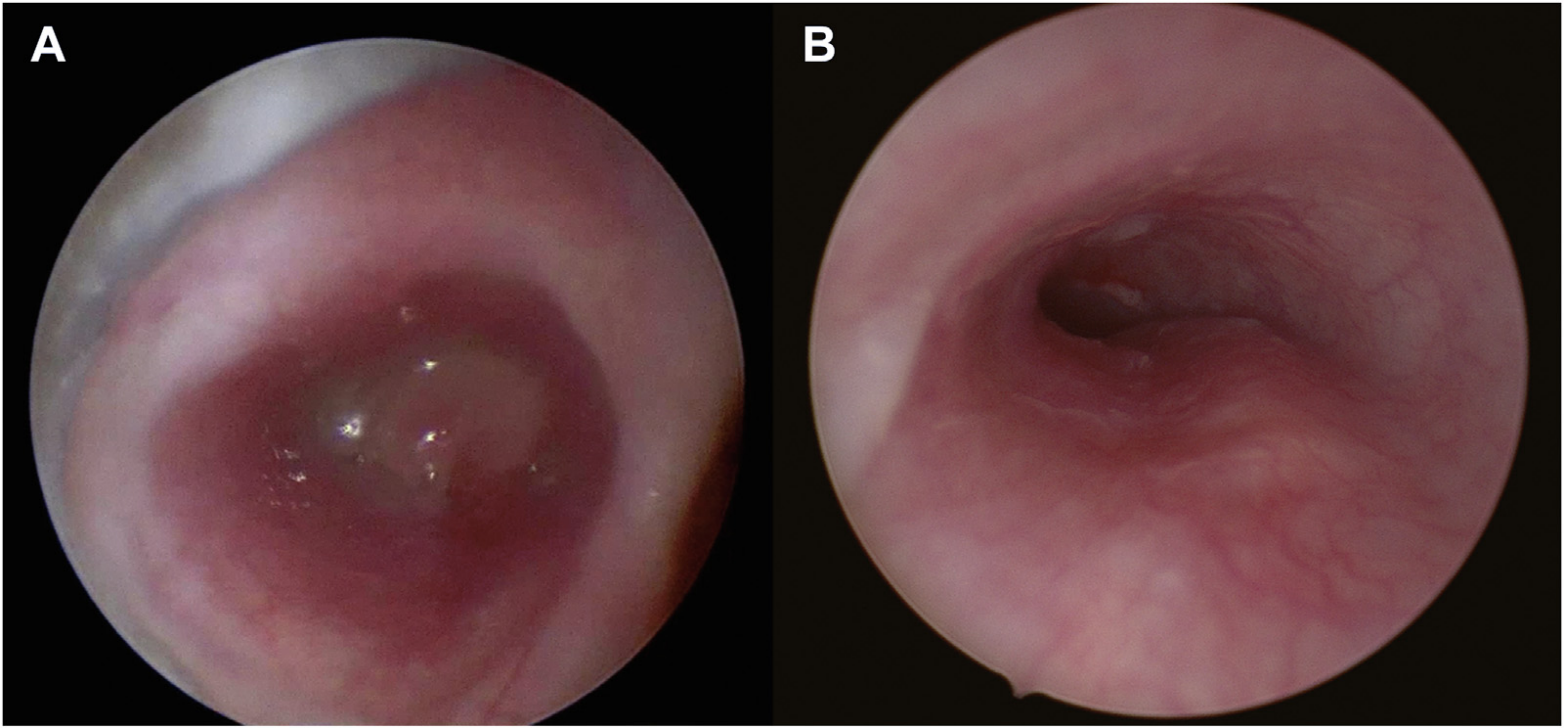
**Granuloma of stent and bronchus remodeling.** (**A**) Left mainstem bronchus with occluding granuloma of stent. (**B**) Bronchus remodeling after stent removal.

All 8 patients have had their stent removed, with a median stent therapy time of 6.8 months (range, 0.4-16.3 months). All stents maintained their radial strength and had the same diameter at placement and removal. Median follow-up since time of stent placement was 15.6 months (range, 7.2-31.3 months), and median follow-up since time of stent removal was 7.6 months (range, 1.6-19.5 months). After stent removal, bronchoscopy demonstrated remodeling of variable degrees of the bronchus into a rounded configuration in the shape of the stent in 6 of 8 patients (Figure 2, Figure 3, Figure 4). Two patients demonstrated return of compressive malacia upon removal, one that proceeded to a pulmonary artery plasty and the other to repeat palliative stenting. There were no bronchial stent erosion events or patient deaths secondary to stenting. One patient developed mild granulation tissue on surveillance bronchoscopy, and 1 patient was noted to have an overlying occlusive granuloma at time of planned stent removal 1 month following a COVID-19 infection but maintained a remodeled shape after removal (Figure 4).

## Discussion

To our knowledge, this represents the first report of bronchial remodeling following placement of balloon-expandable bare-metal stents in pediatric patients with tracheobronchial disease. Our results are encouraging, particularly given our low complication rate and success in stent removal (Central Illustration). Although airway stents are typically only considered in palliative cases or in patients who have failed other treatment options due to their short-term use and complications,^1^ the evidence of airway remodeling in patients who underwent stent retrieval suggests that stents may have previously unrecognized longer-term benefits. This may be particularly useful for CHD patients, as mortality risk is significantly increased among those with CHD and concurrent airway anomalies compared to those with CHD alone.^6^ Because the pathologies of these conditions can both mimic and impact one another, early identification and treatment of airway pathology can be crucial for optimal management of cardiac disease.

**Central Illustration:**
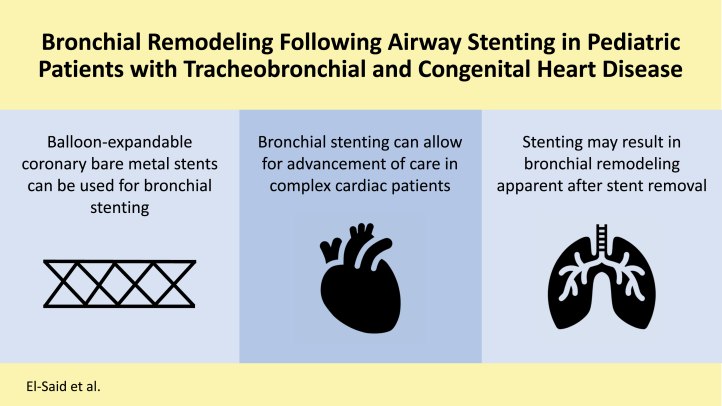
Bronchial stenting and subsequent remodeling. In pediatric patients with concurrent tracheobronchial and congenital heart disease, airway stenting with coronary bare-metal stents resulted in remodeling after stent removal.

However, children with tracheobronchial disease remain a heterogeneous and complex population requiring individualized treatment plans. Malacia and stenosis are the most common etiologies of pediatric tracheobronchial disease and can be divided into congenital and acquired types. Congenital tracheobronchomalacia is caused by an inherent weakness in the cartilaginous rings causing collapse during expiration, and mild to moderate symptoms generally self-resolve with expectant management by the age of 3 years.^2^^,^^7^ If severe, it can be treated with a slide tracheoplasty,^8^ and stents are most useful for postoperative complications in these cases.^9^ Acquired tracheobronchial malacia is weakening of the cartilaginous rings due to an extrinsic factor, most commonly vascular compression, tracheoesophageal fistula, or tracheotomy,^7^ and can be treated with balloon dilation, laser photoresection, or surgical resection. Stenting can be used if surgery fails or is contraindicated.^8^^,^^9^ For children with malacia who cannot be extubated or who have repeated apneic episodes or other severe symptoms, surgical intervention is indicated, most commonly aortopexy, tracheopexy, or surgical relief of external compression. If these measures fail or are contraindicated, tracheostomy or airway stenting are considered next-line options.^9^ For the patients in this report, we used a multidisciplinary approach to deploy airway stents prophylactically or after surgery to allow progression in care. Typically, we chose to stent when there was a single level of airway obstruction or one particularly severe area, which allowed us to avoid a tracheostomy. If there were multiple levels of airway obstruction, particularly in the setting of lung or residual cardiac disease, we opted for a tracheostomy and ventilation if needed. In patients with high respiratory requirements or distress despite tracheostomy, rescue stenting subsequently improved their clinical status.

The timing of stent removal is often a nuanced decision, with a goal of maximizing respiratory support from the stent and minimizing risk of epithelization or granulation tissue making removal difficult. For several of our patients, we left the stent in place until the patient had grown sufficiently to undergo optimal timing of cardiac surgery, at which time we felt the combination of expected airway maturation, de-escalation of respiratory support, and decreased vascular compression rendered the stent unnecessary. In this way, stenting for a relatively short duration may serve as a bridge while the child’s airway matures, allow respiratory support weaning, and possibly allow for favorable remodeling.

### Pediatric bronchial stent types

Currently, no ideal stent exists—each type of stent has individual benefits and drawbacks that must be considered.

#### Balloon-expandable bare-metal stents

Balloon-expandable bare-metal stents are the most commonly used in children, particularly because they can be sequentially dilated as the patient grows.^4^^,^^10^^,^^11^ Ballooning of previously placed stents can also be used to remodel distorted stent structures, compress granulation tissue, and crush a stent for easier removal.^11^ Bare-metal stents rarely migrate and work well for small pediatric airways due to their large internal-to-external diameter ratio.^5^^,^^7^^,^^9^ In addition, they are less likely to impair mucociliary clearance, can be placed over branches as airflow continues through the struts, and are radiopaque so can be seen on chest x-ray and computed tomography.^5^^,^^7^^,^^9^^,^^12^

However, bare-metal stents pose a greater risk of airway or vascular perforation due to their greater expansible force and increased risk of fracturing and erosion into the tracheobronchial wall.^9^ Although rare, severe complications such as bronchial wall perforation or hemorrhage due to arteriobronchial fistula have been reported.^13^^,^^14^ Epithelization or granulation tissue ingrowth through the struts may cause obstruction or make removal difficult, so these stents are not thought to be suitable for long-term use.^5^^,^^7^^,^^9^ Granulation tissue is typically reported as the most common complication and can be mitigated with systemic or inhaled steroids or endoscopic treatment, enabling better results long-term.^9^^,^^11^^,^^15^^,^^16^ If complications arise or the stent is no longer needed, removal of bare-metal stents with rigid endoscopy and forceps is typically attempted.^1^^,^^11^^,^^17^ Retrieval can be a risky procedure, however, with reports of tracheal perforation and granulation tissue obstruction among reasons for reported mortalities.^12^ To mitigate the risks of retrieval, de Trey et al^1^ recommend expanding the stent just sufficiently to leave the stent close to the wall but not snugly against the mucosa to increase the ease of removal. This may slightly restrict the initial lumen diameter but could be a way to avoid future restriction by a nonretrievable stent as the child’s airway grows.^1^ We chose to use the Integrity stent because it has showed lower in-stent restenosis rates in coronary artery disease when compared to other bare-metal stents, and we feel its sinusoidal configuration provides smoother trackability within tortuous anatomy while maintaining its radial strength, allowing precise placement and minimal trauma to the airway during the procedure.^18^

#### Biodegradable stents

To avoid the difficulty of stent retrieval, biodegradable stents have been developed to allow for short-term airway patency and have shown promising clinical results.^8^^,^^9^^,^^19^, ^20^, ^21^ The most common material used is polydioxanone, which retains mechanical strength for 6 to 7 weeks and dissolves after 14 to 15 weeks.^19^ Although rapid absorption allows for growth of the airway, restenting may be required if longer-term patency is needed.^19^^,^^22^ However, in the context of bronchial remodeling we observed in this case series, biodegradable stents may represent an option for longer-term therapy if this remodeling phenomenon extends to these stents as well, particularly for bioabsorbable material with a longer degradation time, such as polycaprolactone.^23^, ^24^, ^25^ However, the longer degradation times of these materials (about 2 years in the case of polycaprolactone) preclude stent upsizing, and the time to degradation may not coincide with remodeling. For these reasons, we chose to use balloon-expandable bare-metal stents, both for their dilation capabilities and the ability to control time of removal when the bronchus and patient were ready.

#### Silicone and other stents

Silicone stents are infrequently used in pediatric patients because their increased wall-to-lumen ratio makes placement in small airways disadvantageous.^2^ When used, silicone stents tend to be firm and durable, are easy to reposition and remove, and incite minimal granulation tissue formation.^7^^,^^26^ However, this ease in movement predisposes silicone stents to migration, and they are also associated with impairment of mucociliary clearance and higher rates of infection.^2^^,^^3^^,^^26^ Other stent types, including hybrid, self-expanding nitinol, and drug-eluting stents, are infrequently employed in pediatric airways due to a paucity of data in this population but merit further research.^2^^,^^7^^,^^9^^,^^27^, ^28^, ^29^

### Limitations

Our sample size of 8 patients is small, and the follow-up time of 15.6 months was relatively short, limiting the generalizability of our study. The etiology of tracheobronchial disease in our patients was heterogeneous, and stents were only considered after initial treatment options failed or were contraindicated. Stent placement was not considered long-term therapy but only as a bridge to definitive therapy or to advance the care of our patients.

## Conclusion

Our airway stenting experience with bare-metal coronary stents in 8 pediatric patients with concurrent tracheobronchial and CHD has produced favorable clinical results and allowed for advancement in care. Furthermore, bronchial remodeling was demonstrated in 6 of 8 patients after stent retrieval, suggesting longer-term benefits than previously thought.

## References

[1] de Trey L.A., Dudley J., Ismail-Koch H. (2016). Treatment of severe tracheobronchomalacia: ten-year experience. Int J Pediatr Otorhinolaryngol.

[2] Pillai J.B., Smith J., Hasan A., Spencer D. (2005). Review of pediatric airway malacia and its management, with emphasis on stenting. Eur J Cardiothorac Surg.

[3] Nicolai T. (2008). Airway stents in children. Pediatr Pulmonol.

[4] Mok Q. (2017). Airway problems in neonates-a review of the current investigation and management strategies. Front Pediatr.

[5] Furman R.H., Backer C.L., Dunham M.E., Donaldson J., Mavroudis C., Holinger L.D. (1999). The use of balloon-expandable metallic stents in the treatment of pediatric tracheomalacia and bronchomalacia. Arch Otolaryngol Head Neck Surg.

[6] Lee Y.S., Jeng M.J., Tsao P.C., Soong W.J., Chou P. (2015). Prognosis and risk factors for congenital airway anomalies in children with congenital heart disease: a nationwide population-based study in Taiwan. PLoS One.

[7] Antón-Pacheco J.L., Cabezalí D., Tejedor R. (2008). The role of airway stenting in pediatric tracheobronchial obstruction. Eur J Cardiothorac Surg.

[8] Stramiello J.A., Mohammadzadeh A., Ryan J., Brigger M.T. (2020). The role of bioresorbable intraluminal airway stents in pediatric tracheobronchial obstruction: a systematic review. Int J Pediatr Otorhinolaryngol.

[9] Antón-Pacheco J.L. (2016). Tracheobronchial stents in children. Semin Pediatr Surg.

[10] Filler R.M., Forte V., Fraga J.C., Matute J. (1995). The use of expandable metallic airway stents for tracheobronchial obstruction in children. J Pediatr Surg.

[11] Hsieh K.H., Chou Y.L., Soong W.J., Lee Y.S., Tsao P.C. (2019). Long-term management and outcomes of tracheobronchial stent by flexible bronchoscopy in infants <5 kg: a 13-year single-center experience. J Chin Med Assoc.

[12] Soong W.J., Tsao P.C., Lee Y.S., Yang C.F. (2018). Flexible endoscopy for pediatric tracheobronchial metallic stent placement, maintenance and long-term outcomes. PLoS One.

[13] Stotz W.H., Berkowitz I.D., Hoehner J.C., Tunkel D.E. (2003). Fatal complication from a balloon-expandable tracheal stent in a child: a case report. Pediatr Crit Care Med.

[14] Miyamoto T., Ishida R., Noma M., Chikada M., Sekiguchi A. (2001). Successful surgical management of a tracheopulmonary artery fistula caused by an intratracheal expandable metal stent. Jpn J Thorac Cardiovasc Surg.

[15] Lim L.H., Cotton R.T., Azizkhan R.G., Wood R.E., Cohen A.P., Rutter M.J. (2004). Complications of metallic stents in the pediatric airway. Otolaryngol Head Neck Surg.

[16] Leung L., Chung P.H., Wong K.K., Tam P.K. (2015). Management of tracheobronchial obstruction in infants using metallic stents: long-term outcome. Pediatr Surg Int.

[17] Mittal N., El-Said H.G., Ratnayaka K. (2021). Bronchial stenting in infants with severe bronchomalacia: technique and outcomes. Int J Pediatr Otorhinolaryngol.

[18] Turco M.A. (2011). The Integrity bare-metal stent made by continuous sinusoid technology. Expert Rev Med Devices.

[19] Vondrys D., Elliott M.J., McLaren C.A., Noctor C., Roebuck D.J. (2011). First experience with biodegradable airway stents in children. Ann Thorac Surg.

[20] Antón-Pacheco J.L., Luna C., García E. (2016). Initial experience with a new biodegradable airway stent in children: is this the stent we were waiting for?. Pediatr Pulmonol.

[21] Sztanó B., Kiss G., Márai K. (2016). Biodegradable airway stents in infants - potential life-threatening pitfalls. Int J Pediatr Otorhinolaryngol.

[22] Griffiths B.T., James P., Morgan G., Diamantopoulos A., Durward A., Nyman A. (2020). Biodegradable stents for the relief of vascular bronchial compression in children with left atrial enlargement. J Bronchology Interv Pulmonol.

[23] Liu K.S., Liu Y.H., Peng Y.J., Liu S.J. (2011). Experimental absorbable stent permits airway remodeling. J Thorac Cardiovasc Surg.

[24] Sun H., Mei L., Song C., Cui X., Wang P. (2006). The in vivo degradation, absorption and excretion of PCL-based implant. Biomaterials.

[25] Wong D.Y., Hollister S.J., Krebsbach P.H., Nosrat C. (2007). Poly(epsilon-caprolactone) and poly (L-lactic-co-glycolic acid) degradable polymer sponges attenuate astrocyte response and lesion growth in acute traumatic brain injury. Tissue Eng.

[26] Fayon M., Donato L., de Blic J. (2005). French experience of silicone tracheobronchial stenting in children. Pediatr Pulmonol.

[27] Donato L.L., Tran T.M., Ammouche C., Musani A.I. (2013). Pediatric interventional bronchoscopy. Clin Chest Med.

[28] Serio P., Fainardi V., Leone R. (2014). Tracheobronchial obstruction: follow-up study of 100 children treated with airway stenting. Eur J Cardiothorac Surg.

[29] Zhu G.H., Ng A.H., Venkatraman S.S. (2011). A novel bioabsorbable drug-eluting tracheal stent. Laryngoscope.

